# Comparison of the Characteristics of Fly Ash Generated from Bio and Municipal Waste: Fluidized Bed Incinerators

**DOI:** 10.3390/ma12172664

**Published:** 2019-08-21

**Authors:** Mudassar Azam, Saman Setoodeh Jahromy, Waseem Raza, Florian Wesenauer, Karolina Schwendtner, Franz Winter

**Affiliations:** 1 Institute of Chemical, Environmental and Bioscience Engineering, TU WIEN, Getreidemarkt 9, 1060 Vienna, Austria; 2 Institute of Chemical Engineering & Technology, University of the Punjab, Lahore 54000, Pakistan; 3 State Key Laboratory of Fine Chemicals, Dalian University of Technology, Dalian 116024, China; 4 Institute for Chemical Technology and Analytics, TU Wien, Getreidemarkt 9, 1060 Vienna, Austria

**Keywords:** heavy metal, fly ash, leaching, hazardous waste, fluidized bed combustion

## Abstract

European solid waste incinerator plants still primarily use grate furnace technology, although circulating fluidized bed (CFB) technology is steadily expanding. Therefore, few investigations have reported on the environmental assessment of fly ash from fluidized incinerators. This research project aims to integrate information on fly ash derived from the combustion of municipal solid waste (FA1) and biomass (FA2) in fluidized bed incinerator facilities. Fly ash samples were comparatively analyzed by X-ray diffraction (XRD), X-ray fluorescence spectroscopy (XRF), scanning electron microscopy (SEM), and inductively coupled plasma optical emission spectroscopy (ICP-OES) to study the mineralogy, morphology, total heavy metal content, and leaching behavior, respectively. The analysis revealed that the two types of fly ash differ in their characteristics and leaching behavior. The concentration of most of the heavy metals in both is low compared to the literature values, but higher than the regulatory limits for use as a soil conditioner, whereas the high contents of Fe, Cu, and Al suggest good potential for metal recovery. The leaching ability of most elements is within the inert waste category, except for Hg, which is slightly above the non-hazardous waste limit.

## 1. Introduction

In recent years, the increase in population and living standards has resulted in an increasing output of municipal solid waste (MSW), which poses a great threat to the environment [1]. The incineration of MSW with energy recovery is an integral part of an environmentally sustainable waste management strategy [2,3]. As most MSW is derived from biomass like waste paper, kitchen garbage, trees and branches, textiles, and leather [4], the extraction of energy via incineration is regarded as a type of renewable energy by the U.S. Department of Energy [5]. An awareness of global environmental issues and the increasing energy demand have been major driving forces in the quest to increase renewable energy sources in Europe’s energy portfolio [6]. The status of fossil fuels and their polluting nature demonstrate the need for new energy technologies that are more efficient and cause minimal environmental damage. The proportion of biomass energy in the world’s total energy consumption is increasing due to the impact of CO_2_ emissions [7]. As a renewable energy source, the combustion of biomass is considered CO_2_-neutral. The CO_2_ produced as a result of combustion is nearly compensated for by the CO_2_ absorbed in the biomass while it grows [8].

In Europe, the emphasis of research is on developing technologies for obtaining renewable energy from biomass to meet the demands of the electricity, heating, cooling, and transportation sectors. These research projects aim to increase the overall efficiency of conversion processes such as combustion, co-firing, and gasification by keeping an eye on cost reduction, environmental impact, and flexibility of technologies to operate under different regional conditions [6,9]. Among these processes, biomass and municipal solid waste combustion by fluidized bed incinerators and grate furnace incinerators are proven technologies for heat and power generation [10]. Fluidized bed incinerators provide good mixing, temperature distribution, high conversion efficiency, fuel flexibility, and low pollutant emissions, but demand higher investment than the primarily used grate furnace technology [9].

Both combustion technologies generate huge amounts of fly ash. The fly ash is problematic for incinerator operation and can cause slagging and fouling in addition to environmental issues. Fly ash contains heavy metals, a high content of easily soluble salts, and, in some cases, polychlorinated dioxins and furans [11,12,13]. Waste management strategies currently recommend disposal in underground deposits or non-hazardous landfill (after stabilization processes) for fly ash. The increase in the cost of development of new landfill sites is of major concern for all energy-generation incinerators, and alternative management of fly ash is being investigated elsewhere [6,9,14].

The quantity, quality, and characteristics of fly ash derived from MSW and biomass depends on many factors, such as the composition of feed, type of incinerator, operating parameters, and pollution control techniques. In Europe, 90% of the incinerators are grate furnaces. The characterization and possible utilization of the bottom and fly ash from grate furnaces have been widely studied, whereas comparative studies of fly ash from fluidized bed incinerators with different feeds are rare [14,15].

This study aims to collect detailed information on the physical and chemical characterization, particle size distribution, mineralogy, morphology, heavy metal content, and leaching behavior of the fly ash generated by two different fluidized bed incinerators, originating from the input feed of municipal solid waste and biomass. This research is part of a major project in which fly ash from different type of incinerators (grate furnace, fluidized bed, and rotary kiln) is being investigated to identify possible utilization opportunities for the fly ash in order to achieve less dependence on landfill [16].

## 2. Experimental

### 2.1. Sampling

The fly ash derived from biomass and municipal solid waste was collected for investigation purposes from two fluidized incinerator facilities. The source feed for biomass incinerator includes forestry residue (wood residue, sawdust, bark, and branches) and agriculture waste. In the case of MSW incineration technology, the feed consists of 85–90% municipal solid waste and 10–15% sewage sludge waste. These plants primarily provide district heating to cities, in addition to power generation. The operating temperature values for MSW and biomass incinerators in beds and freeboard zones ranges between 800 and 900 °C and 1000 and 1100 °C, respectively. In order to get representative samples for lab analysis, fly ash from a cyclone separator and precipitators was collected over a period of two weeks. After grinding and good mixing, the coning and quartering method was repeatedly applied to get suitably sized samples for the different laboratory analyses.

### 2.2. Ash Characterization

Determination of pH and electrical conductivity of the samples was conducted according to European standard SFS-EN 13037 at a solid to liquid (ultrapure water) ratio of 1:5. Determination of the dry matter content of fly ash samples was carried out according to European standard SFS-EN 12880, and fly ash samples were dried overnight to a constant mass in an oven at 105 °C. To determine the organic matter content, measurement of loss on ignition (LOI) was carried out according to European standard SFS-EN 12879. For this, oven-dried (105 °C) samples were heated overnight in a muffle furnace at 550 °C.

Determination of the chemical composition of the fly ash and its fractions was carried out by X-ray fluorescence (XRF) (Panalytical Axios, the Netherlands). The preparation of samples for XRF was done by using 6 g of lithium tetra borate for each 0.5 g of ash, obtained at 1000 °C from the fly ash. The beads were prepared in a platinum crucible under heating and stirring in a Philips Model Perl X3 (PANalytical B.V, the Netherlands). Elemental analysis was performed under standard conditions in a SIEMENS SRS 3000 spectrometer fitted with an Rh target tube.

Powder X-ray diffraction (XRD) (Panalytical, the Netherlands)measurements for mineralogical composition were carried out on a Panalytical Xpert-Pro diffractometer (CuKα, 45 kV, 40 mA, continuous scan, Soller slits 0.04 rad, Bragg-Brentano HD mirror, X’Celerator detector, 2θ range 5°–70°, 200 s/step measurement time). Representative samples were ground manually in an agate mortar for 5–10 min and mounted onto a zero-background sample holder with minute amounts of grease. The evaluation and phase identification were carried out using the search and match routine of the Panalytical HighScore Plus Program Suite [17] on the ICDD database (ICDD, 2017). This was followed by Rietveld refinement with Topas [18] using CIF files from the ICSD database [19]. A Malvern Master sizer 2000 particle size analyzer was used to measure particle size distribution (PSD), with compressed air as the dispersant. Scanning electron microscopy (SEM) analysis was performed on FEI Quanta 200 FEG SEM (FEI, USA), which is equipped with a Schottky emitter in the operating range of 30 to 200 V, supported by an Everhart-Thornley detector for secondary electron in action. In order to decrease the charging fact of the samples to get better results, samples were gold-coated prior to conducting SEM analysis.

For the determination of minor elemental concentration in the fly ash, dried samples were subjected to digestion in aqua regia according to the EN 13657 (2002) standard. Further analysis was done according to the EN 11885 (2009) standard by a PerkinElmer Optima 8300 ICP-OES (inductively coupled plasma optical emission spectroscopy), which was equipped with a SC-2 DX FAST sample preparation system. The analytes were determined via axial view and with three replicates, followed by an arithmetic average. For the calibration, a customized single element (Merck, Roth) standard was adopted. Similarly, leachates were prepared by using a liquid-to-solid ratio of 10 L/kg according to EN 12457-4 (2002). Fly ash with a particle size below 10 mm was used to prepare the leachates in deionized water with continuous tumbler agitation for 24 h. After 10 min of agitation, leachates were subjected to a filtration process (0.45 μm) and subsequent analysis was carried out using the same ICP-OES. 

## 3. Results and Discussion

The physical properties of fly ash are given in Table 1. Low values of LOI of fly ash, even for a shorter residence time as compared to bottom ash, indicate efficient combustion of organic matter in both fluidized bed incinerators. This is due to the temperature range of 820–850 °C in the incinerator bed and 1100–1200 °C in the upper free zone of the incinerator. The electric conductivity of sample FA1 is quite low compared to FA2, showing higher ionic concentration in sample FA2. This means contribution of soluble salts from fly ash samples to conductivity may be taken into consideration for evaluating pozzolanic properties of fly ash samples [20]. The pH of an ash may vary from slightly acidic to highly alkaline, depending on the sulfur content [21]. The pH results show strong alkaline behavior in both samples. An alkaline pH indicates the presence of metal in the ash such as basic metal salts, carbonates, oxides, or hydroxides [22], which is supported by the XRD results. The mineralogical analyses help us understand the coalescent status of the elements in the ash. The toxicity of incinerators solid residues is not only dependent on the concentration of polluting elements, but also on the nature of the host phases [23].

**Figure 1:**
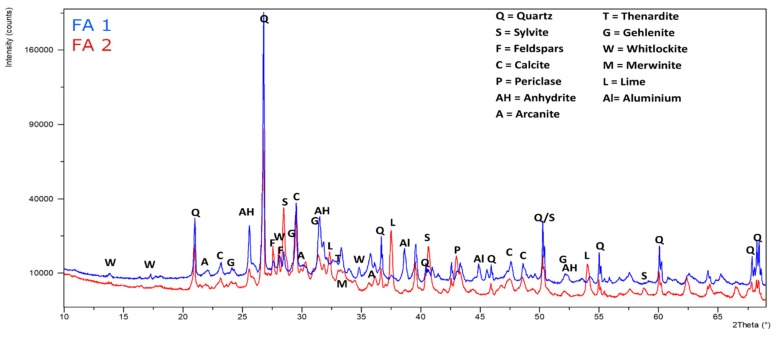
X-ray diffraction (XRD) patterns for fly ash FA1 and FA2.

The X-ray diffraction analysis of crystalline mineral material in fly ash samples is shown in Figure 1 and linked in Table 2. Quartz and calcite are the predominant phases in both samples. Most of the SiO_2_ in sample FA1 is present as quartz, compared to lower amounts in sample FA2. This is explained by the carryover of bed particles of the fluidized incinerator [24] and partially by sand and soil particles in the case of forest residues during harvesting, transport, and handling [25]. Furthermore, there is incineration of plant-tissue-derived Si-based minerals during decomposition, e.g., phytolith (SiO_2_ X nH_2_O), is mostly made up of plant tissue, deposited within and between the plant cells [9]. SiO_2_ is also present in the form of glassy material and other silicate compounds. Another of the major components of forest biomass is Ca [26]. In sample FA2, the calcium concentration is the highest and mainly occurs in the form of calcite and free lime, while in FA1, calcite, anhydrite, and gehlenite are the predominant Ca phases. While most of the sulfate is present as Ca-sulfate in FA1, the high alkali content of FA2 is confirmed by a high content of alkali chlorides (KCl, NaCl), but sulfate is also present, mainly in the form of alkali sulfates (arkanite, thenardite, and aphthitalite). Mg is present as periclase (MgO) in both samples and the results agree with the XRF results. This complex mineralogy is the outcome of many unit operations like melting, vaporization, condensation, crystallization, vitrification, and precipitation, which occur during incineration operation and flue gas treatment [27].

**Figure 2:**
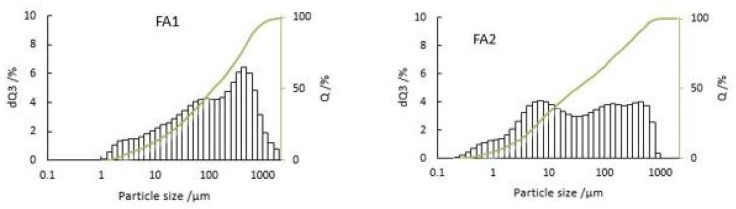
Particle size distribution of fly ash FA1 and FA2.

The particle size distribution of fly ash plays a vital role in assessing and evaluating the potential utilization and environmental impact, as it directly influences the fly ash characteristics [28]. A particle size analysis of fly ash is shown in Figure 2. Sample FA1 is coarser than FA2. According to particle size distribution studies of fly ash, the size may range between 2 and 1000 μm [29]. The D90 of FA1 and FA2 is below 500 and 350 μm, respectively, and D50 is below 100 and 30 μm, respectively, so the PSDs are quite different. In fluidized bed incinerators, high fluidizing velocity is usually maintained to allow the separation of particles in the cyclone segment. Larger particles from cyclone separator are recycled to the main incinerator and fine particles are transported to bag filters. These large recycled particles have a longer residence time in the incinerator, resulting in heavy metal enrichment on the surface of the particles at proper thermal conditions compared to fly ash with fine particles and a short residence time [7,30].

**Figure 3:**
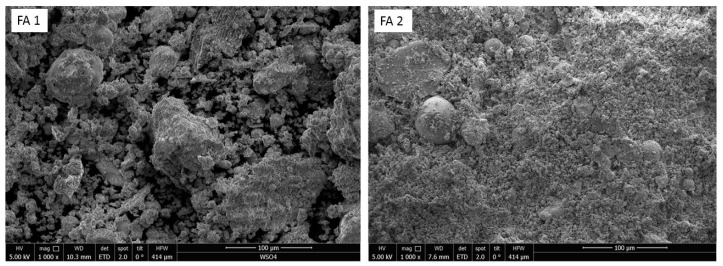
Scanning electron microscopy (SEM) photographs of fly ash samples at 1000× magnification.

The leaching behavior of fly ash depends on the particle properties. For example, the presence of a dense particle interior and porous or non-porous outer surface may affect the rate of leaching of heavy metals [30]. This makes morphology studies of fly ash important. Figure 3 gives SEM photographs of the two samples. The photograph of fly ash FA1 shows large, irregular, and agglomerated particles, which are high-temperature sintering products [27]. Fly ash FA2 photograph shows a fine, homogenous, and partially vesicular structure, which is the result of the volatilization process [31]. It is evident that the surface structure is very different.

According to studies comparing the chemical composition of fly ash, the levels of SiO_2_, CaO, Al_2_O_3,_ Fe_2_O_3_, and MgO in fly ash from fluidized incinerators are higher than those from grate furnace and rotary kiln incinerators. Therefore, the chemical composition of fly ash is greatly influenced by the type of incinerator, input waste, and injection of additives into air pollution control devices (APCD) [29,32]. The major chemical composition of fly ash expressed in the form of oxides, obtained by XRF, is presented in Table 3 and fits well with others’ research results on fluidized bed incinerators using municipal solid waste and biomass as feed [9,29]. SiO_2_ and CaO are the predominant oxides in fly ash [33], making up more than 55% of the total oxide content in both samples. The other main oxides are Al_2_O_3_, MgO, K_2_O, and SO_3_. SiO_2_ and CaO mainly occur as quartz, calcite, and free lime. The CaCO_3_ concentration is a secondary product, as CaCO_3_ will decompose into CaO at the firing temperature. Its presence in a fresh sample could be used to control the firing temperature. Fe_2_O_3_ is present as the mineral hematite in low amounts. The concentration of CaO in biomass fly ash is usually higher compared to bottom ash. This might be due to calcite, which is easily grindable, resulting in a higher CaO content in filter ashes [34]. Moreover, biomass fuel contains calcareous minerals, which also contribute to the CaO content in ash. CaO may also be produced due to thermal decomposition and the subsequent transformation of CaCO_3_ into secondary calcite [35]. This high content of CaO is one of the major reasons for alkaline ash and self-desulfurization in these incinerators [7]. Both the fly ash contained pozzolanic material such as Fe_2_O_3_, SiO_2_, and Al_2_O_3_. For their use in cement industry, the observed low quantity of these materials and presence of chlorides and sulphates in samples can reduce durability of cementitious materials [6].

The enrichment of heavy metals is not only dependent on the concentration of the heavy metals in the fuel. Many other factors, like the presence of heavy metals in bed material and the bed’s age, combustion temperature, ash characteristics, fuel density, and chlorine and sulfur content may contribute as well [36,37]. For example, at 700 °C, bed sand captures the maximum amount of lead (~72%) [38]. It is worth noting that, during the incineration process, physical and chemical properties such as saturated vapor pressure and boiling point are of great importance regarding the volatilization process of heavy metals. The concentration of heavy metals is greatly influenced by the operating temperature of the incinerators. The fluidized bed incinerators’ temperature of 800–1200 °C is high enough to vaporize elements, but the retention time of elements in the fly ash due to other processes like condensation, physical adsorption, and chemical absorption determines the final aspect of volatilization regarding specific elements [39]. Most of these elements condense on the surface of fine particles during flue gas treatment, resulting in the enrichment of some heavy metal elements in the fly ash. Due to this process, the bottom ash mostly consists of non-volatile components with sintered and melted particles.

One notable element is cadmium with a boiling point of 767 °C, which is prone to partial evaporation and condensation on fly ash particles, followed by partial carryover in the gaseous phase to the atmosphere during the incineration process [40]. The use of solid residue from incinerators as a soil conditioner in forestry is ecologically beneficial, as it will improve the level of primary resources. This practice will result in the sustainable utilization of ash from incinerators. However, the sensitivity of physical and chemical characteristic of fly ash with respect to different factors like plant species, growing rate, size and age of trees, collection, storage, incineration technology, operating temperature, and flue gas treatment make it different every time for use as a soil conditioner [41]. Therefore, caution should be employed concerning the use of fly ash in the natural environment and the exact process conditions of the fly ash must be known [42]. The heavy metal contents of the samples are given in Table 4. This shows that both types of fly ash can be deposited in normal landfills. Both samples contain a high level of Cd with respect to their use as a soil conditioner, as mentioned in the Landfill Ordinance [43], but the level is still low compared to the literature data on different fluidized incinerators [44,45]. The phenomena of chemical absorption should be taken into account for the formation of in-volatile Cd compounds on the particle surface [46]. It is worth noting that, in addition to Cd, the concentration of metals like Mo, Cr, Sn, and Sb is notably low, whereas the concentration of Ni, Al, Cu, Zn, and Fe is high with respect to the reported literature data. This shows the good potential for metal recovery for the fly ash. Similarly, the concentration of other metals like Pb, Ba, and Hg, which are essential to monitor before their use as a soil conditioner, is higher compared to the maximum allowable regulatory limits. At present, various methods such as pyrometallurgical, hydrometallurgical, and bio-hydrometallurgical are in use for the recovery of heavy metals. The pyrometallurgical recovery consists of thermal treatment such as roasting, calcining, or smelting, whereas hydrometallurgical recovery mainly consists of a leaching process. Furthermore, the extension of the leaching process (acidic) of fly ash is known as the FLUREC process, which allows for the extraction of Zn, Cd, Cu, and Pb. However, at current Zn prices, this process is only economically viable with fly ash containing Zn content above 50,000 mg/Mg [14]. Metals like Zn, Pb, Cu, and Cd can also be easily recovered during the thermal treatment of fly ash [47]. The use of a microbiological leaching process for metal recovery is a promising bio-hydrometallurgical concept, and could play a major role in the metal recovery system. After recovery, these metals can be utilized as a secondary raw material in the metal industry [48,49,50].

The MSW and solid residue (ash) from incinerators are exposed to different weather conditions and natural processes during storage, utilization, or disposal. This may result in the contamination of different water sources, especially ground and surface water, due to leaching processes. To evaluate the contamination potential of the solid residues given the surrounding environment, different leaching tests are available. These tests have different liquid to solid ratios and methods of pH control. The liquid to solid ratio directly influences the concentration of heavy metals, whereas the leaching character of heavy metals is strongly pH-dependent. Generally, a low pH favors the leaching process of heavy metals. The results of leaching tests based on different pH values or liquid to solid ratios therefore cannot be directly compared, and different standards have been developed for simulating different leaching environments. These tests measure the mobility of heavy metals and provide good insight about the possibilities of their use and treatment before disposal according to the regulatory limits [7,51,52,53].

Table 5 gives the results of leaching tests for both samples with respect to Landfill Ordinance. The limit values are the lower and (in brackets) highest limits for non-hazardous waste landfill as set by the Landfill Ordinance value for solid waste [43]. The results indicate no serious leaching of heavy metals during leaching tests. It is worth noting that the leaching contents of metals like Pb, Cd, Ni, Cu, Zn, Sb, Mo, Sn, Co, and As in both types of fly ash are well under the non-hazardous waste category limit. The leaching level of metals like Cr and Ba in the biomass sample is quite high in comparison to the municipal solid waste sample, but still below the non-hazardous waste limit value. The leaching level of Hg in MSW is slightly higher than the limit for non-hazardous waste, whereas for the biomass sample it is well within the inert waste limits.

## 4. Conclusions

This work studied the detailed characterization of fly ash derived from the fluidized combustion of municipal solid waste (FA1) and biomass (FA2). The particle size distribution, chemical composition, morphology, mineralogy, and leaching behavior of heavy metals from samples of fly ash from two fluidized beds were assessed for comparative characterization studies and led to the following conclusions.

- Fly ash FA1 (100–500 μm) at D90 and D50 is coarser than FA2 (30–350 μm) and SEM analysis clearly found that the two types of fly ash have different surface structures. This means both fly ash samples will execute different filling, surface, and water affinity or lubrication role for their potential applications.
- XRD analysis demonstrates a complex mineralogy in which quartz and calcite are the major components. The high amounts of alkalis are present in the form of chlorides (sylvite) and sulfates (arkanite, thenardite, and aphthitalite) in FA2, while the sulfate is concentrated as anhydrite in FA1. Mg is mostly present as periclase and merwinite. The amorphous phase content seems to be low (<20%); therefore, the pozzolanic activity is estimated to be low.
- XRF analysis shows higher amounts of SiO_2_, Al_2_O_3_, and Fe_2_O_3_ in FA1, while the levels of CaO, K_2_O, and MgO are higher in FA2.
- Inductive couple plasma (ICP) analysis clearly showed that the heavy metal concentration for most of the metals is within the literature values. The heavy metal concentration for both types of fly ash is higher than the regulatory limits for their use as a soil conditioner. However, the high levels of Fe, Cu, Al, and Ni indicate their potential for the metal recovery process.
- The leaching test showed no serious leaching for both types of fly ash. Leaching levels for most of the metals are good within the inert waste category, except for Hg in FA1, which is slightly above the non-hazardous waste category. The leaching levels of Cr and Ba in FA2 are higher than FA1 but below the non-hazardous waste category.

## Figures and Tables

**Table 1:** General characteristics of fly ash.

Parameter	FA1	FA2
Fuel type	MSW	Biomass
Ash type	ESP, Cyclone	Bag filters
Dry matter content (105 °C)	98.44	98.66
pH	11.75	13.25
LOI (550 °C)	3.1	1.8
Electrical Conductivity (mS/cm)	5.92	25.78

**Table 2:** Mineralogical analysis (XRD) of fly ash samples.

Mineral Phases		FA1	FA2
Quartz	SiO_2_	+ + +	+ +
Anhydrite	CaSO_4_	+ +	+
Calcite	CaCO_3_	+ +	+ +
Lime	CaO	-	+
Periclase	MgO	+	+ +
Sylvite	KCl	+	++
Halite	NaCl	-	-
Gehlenite	Ca_2_Al_2_SiO_7_ )	+ +	+
Merwinite	Ca_3_Mg (SiO_4_)_2_	+	-
Feldspar	(Ca, Na, K) (Al, Si)_4_ O_8_	+	+ +
Whitlockite	Ca_3_ (PO_4_)_2_	+	-

+ + + High intensity, + + Medium Intensity, + Low intensity, - Not detected.

**Table 3:** Comparison of inorganic fraction of fly ash.

Element	FA1 (wt %)	FA2 (wt %)
Na_2_O	2.6	2.1
MgO	2.1	6.1
Al_2_O_3_	20.7	8.2
SiO_2_	38.3	34
P_2_O_5_	4.2	2.7
SO_3_	2.2	6.0
Cl	1.5	2.9
K_2_O	1.2	7.9
CaO	18.5	23.4
TiO_2_	1.2	1.3
Fe_2_O_3_	4.4	2.9
Organic dry matter	2.7	1.9

**Table 4:** Total heavy metal concentration in fly ash samples (mg/kg, dry weight basis).

Metal	FA1 (Mean)	SD	FA2 (Mean)	SD	Landfill Limit Value [43]
Al	65,583	1040	26,778	434	-
Fe	31,124	389	15,939	776	-
Zn	5363	53	10,001	145	-
Pb	1529	17	1528	59	-
Cu	1941	38	603	4.2	-
Ba	865	10	2172	116	-
Cr	159	1	91	1.6	-
Sn	92	11	83	9	-
Ni	88	1.4	86	0.8	-
Hg	67	3.2	1.12	0.08	20
Sb	67	7.5	122	9.8	-
Co	30	0.2	30	0.63	-
Cd	9.5	0.18	16	1.5	5000
Mo	7.8	0.8	7.2	0.68	-
As	6.3	0.38	16	2.5	5000

SD: standard deviation.

**Table 5:** Leaching capacity of heavy metal content from ash using EN 12457-4 (2002).

	Leaching Capacity (mg/kg)				Limit Value (mg/kg) [43]
Metal	FA1 (Mean)	SD	FA2 (Mean)	SD	Non-Hazardous Waste
Pb	<0.036		0.381	0.04	10 (30)
Cd	0.005	0.01	<0.0018		1
Ni	0.004	0.01	0.018		10
Cr	0.965	0.02	2.43	0.02	10 (20)
Cu	<0.003		0.11	0.01	50
Zn	<0.003		0.86	0.02	50 (100)
Sb	0.062	0.03	0.073	0.02	
Mo	0.167	0.01	0.287		
Hg	0.36	0.04	0.001		0.1
Sn	0.126	0.06	<0.0153		20
Fe	<0.0051		0.031	0.04	-
Co	<0.009		<0.009		5
Ba	0.401	0.01	2.31	0.21	100 (300)
As	0.019	0.01	0.016	0.01	2
Al	<0.0051		4.25	1.01	-

SD: standard deviation.
